# Subtle morphological changes in the visual and antennal sensory system of bees and wasps across an urbanisation gradient

**DOI:** 10.1038/s41598-024-58804-2

**Published:** 2024-04-18

**Authors:** Andrea Ferrari, Greta Tacconi, Carlo Polidori

**Affiliations:** Department of Environmental Science and Policy (ESP), University of Milan, Via Celoria 26, 20133 Milan, Italy

**Keywords:** Eyes, Sensilla, Sensory system, Urbanisation, Wasps, Wild bees, Urban ecology, Entomology

## Abstract

Increased temperature and fragmentation of green spaces in urban areas could drive variations in functional traits of insects. Such morphological shifts may occur for sensory systems, which were previously reported to be prone to change with habitat characteristics in non-urban contexts. Here, we measured traits related to the visual and antennal sensory systems in the bees *Halictus scabiosae* and *Osmia cornuta* and the wasp *Polistes dominula* along an urbanisation gradient within Milan (Italy). We hypothesised that fragmentation could filter for better visual properties, and that higher temperature could filter for fewer thermoreceptors and more olfactory hairs. While controlling for body size, results show subtle but appreciable responses to urbanisation in one or more traits in all species, though not always supporting our hypotheses. *O. cornuta* shows marginally higher ommatidia density and smaller ommatidia diameter (associated with better visual resolution) in more fragmented sites, as well as marginally fewer thermoreceptors in hotter sites, in agreement with our two predictions. On the other hand, *H. scabiosae* has marginally smaller antennae and* P. dominula* has smaller eyes at warmer locations, and the wasp also has smaller antennae and 9th flagellomeres in more fragmented areas. Perhaps higher temperatures accelerate development of sensory system at higher speed than the rest of body in these two species. Our results represent the first evidence of urbanisation effects on the visual and antennal sensory systems of bees and wasps and underline how such effects may involve a much broader bouquet of traits then previously observed.

## Introduction

The growth in human population is leading to an increase in land-use change. One of the main consequences is the replacement of natural cover with cemented surfaces, a phenomenon known as urbanisation^1^. Urbanisation leads to increasing temperature and fragmentation of green areas. The rise in temperatures in cities, known as the Urban Heat Island effect, occurs because of the loss of tree cover and the increase in cemented surfaces that act as heat sinks^2^. In addition, buildings, roads, and other infrastructure fragment green areas so that highly urbanised landscapes are typically characterised by scattered patches of green cover^1^.

Urbanisation not only affects the landscape, but also the flora and fauna therein. For example, we are witnessing a decline in insect diversity and biomass^3^, which, at least for certain groups, is partially due to increased urbanisation^4,5^. Among the insect groups which attracted attention in recent urban studies, bees and wasps are certainly relevant, as they provide important ecosystem services such as pollination^6^ and pest control^7^. Due to their importance and the relatively high abundance and diversity in urban areas^8,9^, community-level diversity studies—and to a lesser extent—functional ecology studies, are being performed across cities around the world^8,10–12^. Still, substantial knowledge about how urbanisation could drive morphological or physiological variations in these organisms is still lacking^13,14^, with most studies focusing almost exclusively on body size variations^15,16^. Among the target morphological traits that merit attention there are those associated with sensory system, whose possible variation in urban habitats has never been investigated. However, we can make hypotheses regarding the possible effects of urbanisation (UHI effect and increased fragmentation) by drawing on what has been done in laboratory experiments or has been studied along natural gradients(Ref.^14^ and references therein), as highlighted below.

The sensory system of bees and wasps is mainly composed of the visual apparatus (two compound eyes and three ocelli) and the antennal apparatus (two antennae covered with sensilla). Compound eyes are responsible for image formation and are composed of thousands of ommatidia, their functional units^17^. Each ommatidium consists of an optical part and a sensory part, which transforms the captured light into an electrical signal. The balance between size of the eye, ommatidia and their density shape the visual properties of the compound eye. In fact, increased resolution is achieved with bigger eyes, since the size of the eye increases the resolution^17^. Enlarged eyes permit a general improvement of both spatial resolution and contrast sensitivity^18^. In addition, better resolution is also obtained by reduced interommatidial angle (i.e., the angle between the optical axes of adjacent ommatidia) or higher ommatidia density^19^. On the other hand, bigger eyes, bigger ocelli, and larger ommatidia are associated with an increased light capture^17^. The antennal sensilla are cuticular structures that contain receptors for the perception of different stimuli and have a range of morphologies that underlines such different functions^20^. In bees and wasps, there are several types of antennal sensilla. Pit organs (PO) which have a thermal- (cold sensitive), hygro- and/or CO_2_-detecting function, sensilla placoidea (SP) and basiconica (SB) which have an olfactory/gustatory function, and sensilla trichoidea (ST) which have either an olfactory, gustatory or mechanoreceptor function, depending on the sub-type (see^21–24^).

Much effort has been devoted to understanding the evolution of sensory systems in insects, and we now have a large bulk of evidence that both visual and antennal apparatus differ between species and between sexes within species in function of the variability of life-history traits such as flying time, food specialisation, foraging strategies, mating tactics and social behaviour^18,20,22–29^. More relevant to the present study, insect sensory system was also found to change morphologically across environmental gradients. For example, Kierat et al.^30^ found that the ommatidia size of a solitary bee of the genus *Osmia* decreases with increasing temperature. In addition, larger eyes and larger ommatidia size were found in butterflies inhabiting naturally more fragmented areas^31^. Moreover, Boulton and Field^32^ found that in warmer locations the bee *Halictus rubicundus* (Christ, 1791) have more olfactory hair density and less hygro-thermal receptors, this latter result being likely due to the cold-sensitive nature of thermal receptors. Finally, the antennal sensillar apparatus of triatomines (Hemiptera) showed morphological variation among populations living in different habitats, with urban specimens showing a pronounced variability and lab-reared specimen a low variability^33^.

Here, we investigated for the first time how some morphometric traits of visual and antennal sensory systems vary according to urbanisation level (here characterized by temperature and fragmentation of green areas) in three aculeate Hymenoptera: the bees *Halictus scabiosae* (Rossi, 1790) and *Osmia cornuta* (Latreille, 1805), and the wasp *Polistes dominula* (Christ, 1791). Based on the above-cited evidence on the relationship between sensory system and climatic and habitat characteristics, we formulated the following non-mutually exclusive hypotheses. First, greater fragmentation of green areas could filter for an eye morphology which improves resolution and/or light capture, as these would improve the insect's ability to move in a fragmented environment^31^. In addition, eye size may be reduced in warmer sites following the theory of the optimal cell size: higher temperatures require a higher demand for oxygen which favours tissues built of small cells^30,34^. Second, in hotter sites we expect lower numbers of hygro-thermal receptors, since they are cold-sensitive receptors, and/or higher densities of olfactory/tactile hairs^32^ and smaller antennae, following the allometric scaling of these appendages with body size, together with the reduction of body size with increasing temperatures^35,^ and an overall fast larval development^36^. Finally, since in holometabolous insects the eyes and the antennae are believed to arise from the same imaginal disc^37^, we may expect a trade-off in the allocation of the resources between these structures. Indeed, visual and olfactory/tactile stimuli could be differently important for individuals living in different environments along the urbanisation gradient^29,37,38^.

## Materials and methods

### Studied species and sampling activities

The study was performed in the metropolitan city of Milan (45°28′01″ N; 9°11′24″ E) and the nearest semi-natural outskirts situated in Lombardy, northern Italy. We selected a total of 18 sites (Table S1, Fig. S1A) along an urbanisation gradient. The site selection was based on the amount of impervious (i.e., cemented) surface to create a gradient of urbanisation. Sampling sites were separated by more than 1 km as this would approximate a plausible maximum foraging distance of the species analysed^39^. The field work took place between March and June 2022. We chose three aculeate Hymenoptera species abundant in cities, morphologically easy to identify and with different ecological traits. *O. cornuta* is a solitary, polylectic bee species that nests in pre-existing cavities^40^. Conversely, *H. scabiosae* is a primitively eusocial, ground-nesting and polylectic bee species^41^. Finally, *P. dominula* is a eusocial paper wasp that builds nests from chewed wood fibres and is a generalist predator^42,43^ (Fig. S1B). Insects (all females) were hand-netted, placed in 1.5 mL plastic vials and stored in cool-bags in the field, and then stored in the lab at − 20 °C for the morphological analyses.

Overall, 40 individuals of *O. cornuta* from 8 sites, 65 individuals of *H. scabiosae* from 13 sites and 47 individuals of *P. dominula* from 10 sites were analysed in the study (Table S1).

### Landscape characterisation

We followed the method presented in^44^ for the landscape characterisation. All variables were calculated in never overlapping 500 m buffers; thus, the environmental variables were never shared among sites. We characterised each site in terms of temperature and fragmentation of green areas. Temperature was estimated using MOD11A2 (https://modis.gsfc.nasa.gov/data/dataprod/mod11.php) downscaled from the original resolution of 1 km to 100 m trough bilinear interpolation. Because different morphologies in adult insects largely depend on conditions during the development, temperature values used here refer to those affecting the sampled individuals during their development periods (which vary among the three species), and not the temperature occurring during the sampling days (see^44^ for details). We decided to use the temperature across an extended period of time since longer periods buffer any possible punctual fluctuations in temperature that often occur from week to week.

Edge density was calculated as the ratio between the total perimeter and surface of green patches. This is one of the ways to quantify the degree of fragmentation of green areas, where higher edge density values indicate more fragmented green areas. The metrics of green areas were obtained from DUSAF6.0 (https://www.dati.lombardia.it/Territorio/Dusaf-6-0-Uso-del-suolo-2018/7rae-fng6). It contains a land-use map of the entire region Lombardy with a resolution of 20 m. Within DUSAF there are 98 land-use classes (https://www.cartografia.regione.lombardia.it/metadata/Dusaf/doc/Legenda_DUSAF_2018_6_0.pdf) that were summarised in two main categories (e.g. ^44^): impervious surfaces (i.e., all paved or build areas, roads, or railways) and green areas (i.e., areas covered with vegetation, both natural and cultivated). We checked for spatial autocorrelation using the Moran’s test^45^, excluding possible autocorrelation of temperature and green fragmentation (all *P*-values > 0.05).

### Body size

We first measured the intertegular distance and the head width as proxies of the body size of the insect through a LEICA MZ75 stereomicroscope mounted with a LEICA flexacam C3 camera (accuracy 0.001 mm). Then, we took all the measures associated with the visual and antennal sensory system, as detailed below.

### Visual sensory system

**Figure 1:**
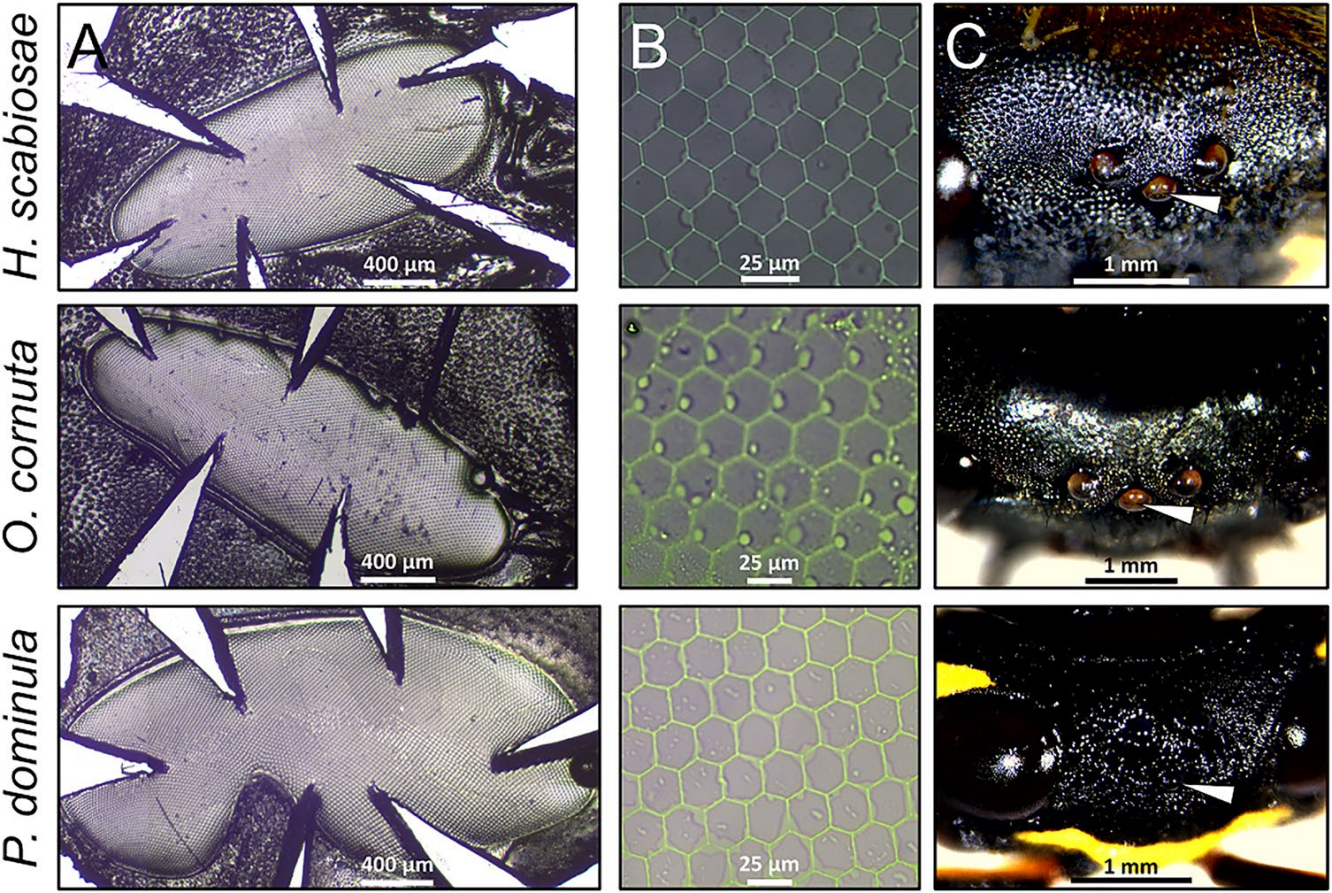
Examples of (**A**) a nail-polish replica of an eye, (**B**) magnifications of the eye replicas and (**C**) of heads (with arrowhead pointing to the median ocellus) for the three species.

We produced a nail-polish replica of one compound eye for each specimen using a transparent topcoat nail polish (denser) and a transparent standard nail-polish (more fluid). To do so, we firstly applied, with a thin paint brush, a layer of a 1:1 mixture of topcoat and nail-polish over and around the eye to remove the facial hairs. We applied the layer for 7 min and replicated this operation three times to completely shave the face of the specimen. Then, we applied the last very thin layer of pure nail-polish for 20 min just on the compound eye and then gently removed it using a scalpel to detach the replica and forceps. We placed the replica on a microslide and made some incisions around its border with the tip of a scalpel perpendicular to the replica, without eliminating fractions of the eye surface (Fig. 1A). With this step, we were able to flatten the replica (otherwise being curved), cover it with a coverslip and seal the slide with nail polish. We let it dry overnight before taking the pictures at the stereomicroscope. Such method was previously used to analyse eye morphology of bees and wasps^18,46^.

We firstly photographed the whole replica to measure the total area of the compound eye. Then we took three random non-overlapping photographs of the central portion of the eye (to avoid the incised parts) to have the ommatidia at an adequate magnification for the measurements (Fig. 1B). For the analysis, we used the average between the measures on the three non-overlapping images. We counted the number of ommatidia and calculated their density dividing this number by the area of the image. Likewise, we estimated their total number knowing the area of the eye. With this number we calculated the interommatidial angle as $\Delta \gamma$ = (23,818/ommatidia number)^1/2^^47^. We also measured the diameter of 10 randomly chosen ommatidia for each image with which we calculated the mean diameter (i.e., proxy of their size). Finally, we measured the diameter of the median ocellus through the stereomicroscope (Fig. 1C).

### Antennal sensory system

**Figure 2:**
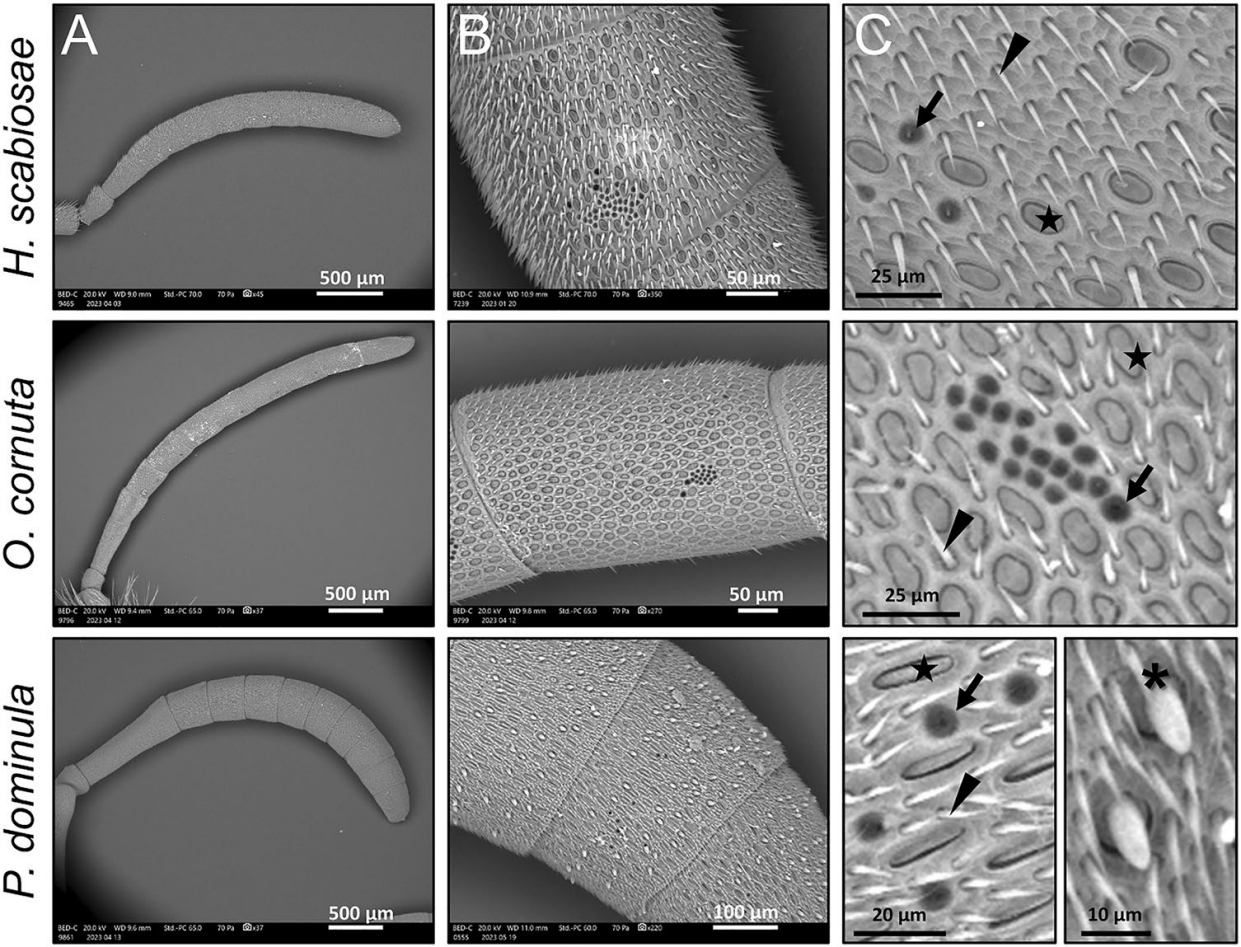
(**A**) Examples of a SEM images of the whole antenna of the three species. (**B**) SEM images of the F9 of the three species. (**C**) Examples of sensilla trichoidea (arrowhead), thermoreceptors (arrow), sensilla placoidea (star) and sensilla basiconica (asterisk) in *P. dominula* in the three species. Note that sensilla basiconica were only found in *P. dominula*.

We detached both antennae from each specimen and placed them on pin stubs, taking care that they fell sideways, each one on a different side (i.e., right and left). For each antenna, we took a photograph through a JEOL JSM-IT500 LV Scanning Electron Microscope (SEM) (JEOL Ltd., Tokyo, Japan), equipped with Backscattered (BSE) and Secondary Electrons (SE) detectors. The operating conditions were: vacuum mode, 20 kV accelerating voltage and 10 mm working distance. We pictured both the entire antenna (Fig. 2A) and the 9th flagellomere (hereafter, F9), the one preceding the most distal one, known to have the highest density of sensilla^22,23^ (Fig. 2B). For the data collection, we randomised right or left among individuals. From the SEM images we measured the total length of the antenna and length and width of F9 (from which we obtain its area, assuming its shape as a rectangle). We recognised four different types of sensilla with the same morphology in the three species (Fig. 2C): pit organs (PO), sensilla trichoidea (ST) and sensilla placoidea (SP) shared by the three species, and sensilla basiconica (SB) found only in *P. dominula*. All these types of sensilla are distinguishable from their morphology. PO are round pores on the surface of the flagellomere, ST are quite long and perpendicular to the antennal surface, with a sharp end, SP are plates with rounded edges shaped into grooves, with the surface slightly depressed in relation to the body wall level, while SB are straight hairs with a blunt tip and a single pore, which sometimes may not be visible. We did not distinguish further sub-types of ST^21–24^ as these are not relevant for the purpose of this work. While the PO are known to include not only thermo-hygro receptors but also CO_2_ receptors, the relative role of these stimuli is still unclear for these sensilla^48–50^. Hence, we decided to consider them as a single category of climate-responding sensilla, as in^32^. On the F9, we counted the total number of thermo-hygro receptors (PO). Then, in ImageJ, we placed on the F9 a square of 100 μm^2^ where the highest density of sensilla was present and—in that square—we counted the number of ST, SP and SB.

### Statistical analysis

All the analyses were performed in R 4.2.2^51^. Firstly, we checked with a Pearson test that the proportion between green and impervious surface correlates with both temperature (*r* =  − 0.517, *P* = 0.028) and green edge density (*r* =  − 0.527, *P* = 0.025). We produced a correlation matrix between all the morphological traits measured for each species to visualise how they co-vary. In addition, we produced the same matrix but with weighted morphological parameters (i.e., after having divided them by the intertegular distance). With this second matrix we could assess possible allometric scaling of the sensory system with body size.

Then, the analysis was done using Linear Mixed Models, using the sampling site as a random effect to account for the interdependence of samples and the intra-population variability. In all models, we used only the intertegular distance as a proxy of body size (since it was strongly correlated with head width, *r* = 0.936, *P* < 0.001) to account for the intraspecific variability and the effect that body size has on the tested parameters. According to our hypotheses, visual and antennal traits may vary with temperature and/or green edge density. Hence, we selected the best model using a theoretical information approach (Bishop et al. 2016). We predict the best model to be the one to minimise the bias corrected Akaike information criterion (AIC_c_) while maximising marginal (due to fixed effects only) and conditional (due to fixed effects and random effects) R^2^^52,53^. We also evaluated possible non-linear responses by transforming the environmental predictor either via logarithm or a square power. If this transformation did not improve the model, we kept the linear response. Finally, for each model, we confirmed that the assumptions of residual normality^54^ and homoscedasticity^55^ were always met with the function “check_model()” from the package *performance*^56^. Other R packages used were ggplot2^57^ and corrplot^58^ for graphics, *lme4*^59^ and *sjPlot*^60^ for mixed models.

## Results

**Figure 3:**
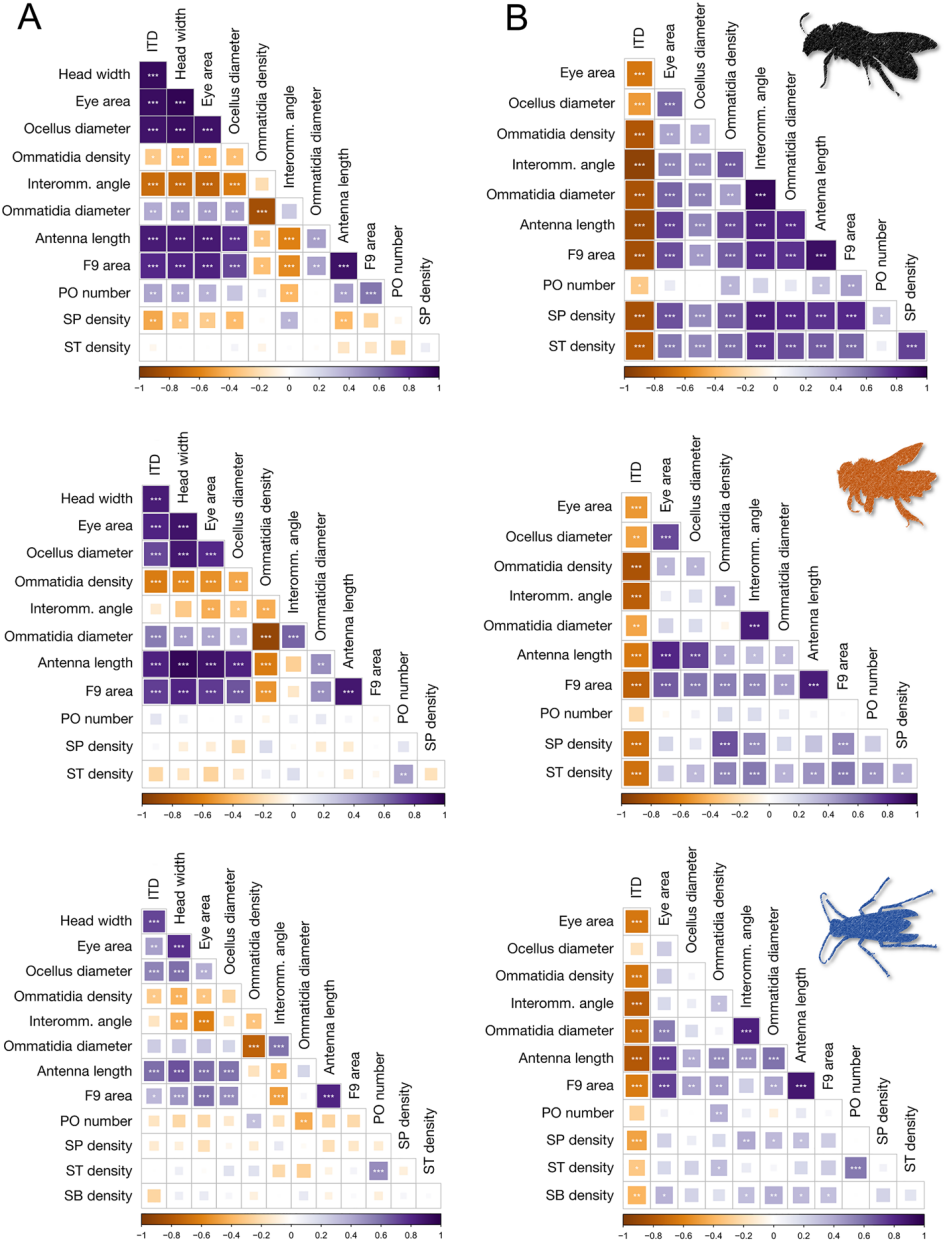
(**A**) Correlation matrices (Pearson’s r) between the morphometric parameters measured on each specimen for the three species*.* (**B**) The same matrix but the morphometric parameters are weighted by body size (i.e., intertegular distance). Colours represent the correlation (r values in the scale below the matrix), in each box the significance of the correlation is reported: *** < 0.001, ** < 0.01, * < 0.05. ITD: intertegular distance, Interomm.: interommatidial, PO: pit organs, SP: sensilla placoidea, ST: sensilla trichoidea, SB: sensilla basiconica.

Morphometric parameters associated with both visual and antennal sensory systems (Table S2) were variably correlated between each other (Fig. 3A). As expected, in the three species there was a strong positive correlation between the body size (both as intertegular distance and head width) and the size of the primary structures of the two systems: the eye area and the length of the antenna (Fig. 3A). In turn, these two main variables were correlated with the other parameters of the respective sensory system. Even at this more detailed scale, albeit less consistently, we could find common patterns among the three species. We found that the eye area was positively correlated with the diameter of the ommatidia and negatively correlated with the interommatidial angle and the density of ommatidia (Fig. 3A). For the antennal sensory systems there were weaker patterns. The area of the 9th flagellomere was strongly correlated with the length of the antenna, but the number of the various sensillar types were not strongly correlated with the length of the antenna (Fig. 3A). On the other hand, co-variation between components of visual and antennal system was rarer (Fig. 3A). Among the significant correlations between other parameters from the two sensory systems, we found that the parameters regarding the ommatidia significantly correlated with antennal length and/or F9 area (Fig. 3A). In general, much weaker correlations—or no correlations at all—were found between the number or density of individual sensilla types and visual parameters (Fig. 3A).

We also visualised how the morphometric parameters correlate between each other and differ between species when weighted by the intertegular distance (Fig. 3B and S2). Consistently in the three species, the weighted parameters were negatively correlated with the body size (Fig. 3B). This means that in larger insects the structures of the sensory systems are smaller than expected by an isometric relationship. Finally, most of the structures of the sensory systems are positively correlated between each other when weighted by body size (Fig. 3B). This is particularly true for *H. scabiosae* and less evident in *O. cornuta* and *P. dominula* respectively.

**Figure 4:**
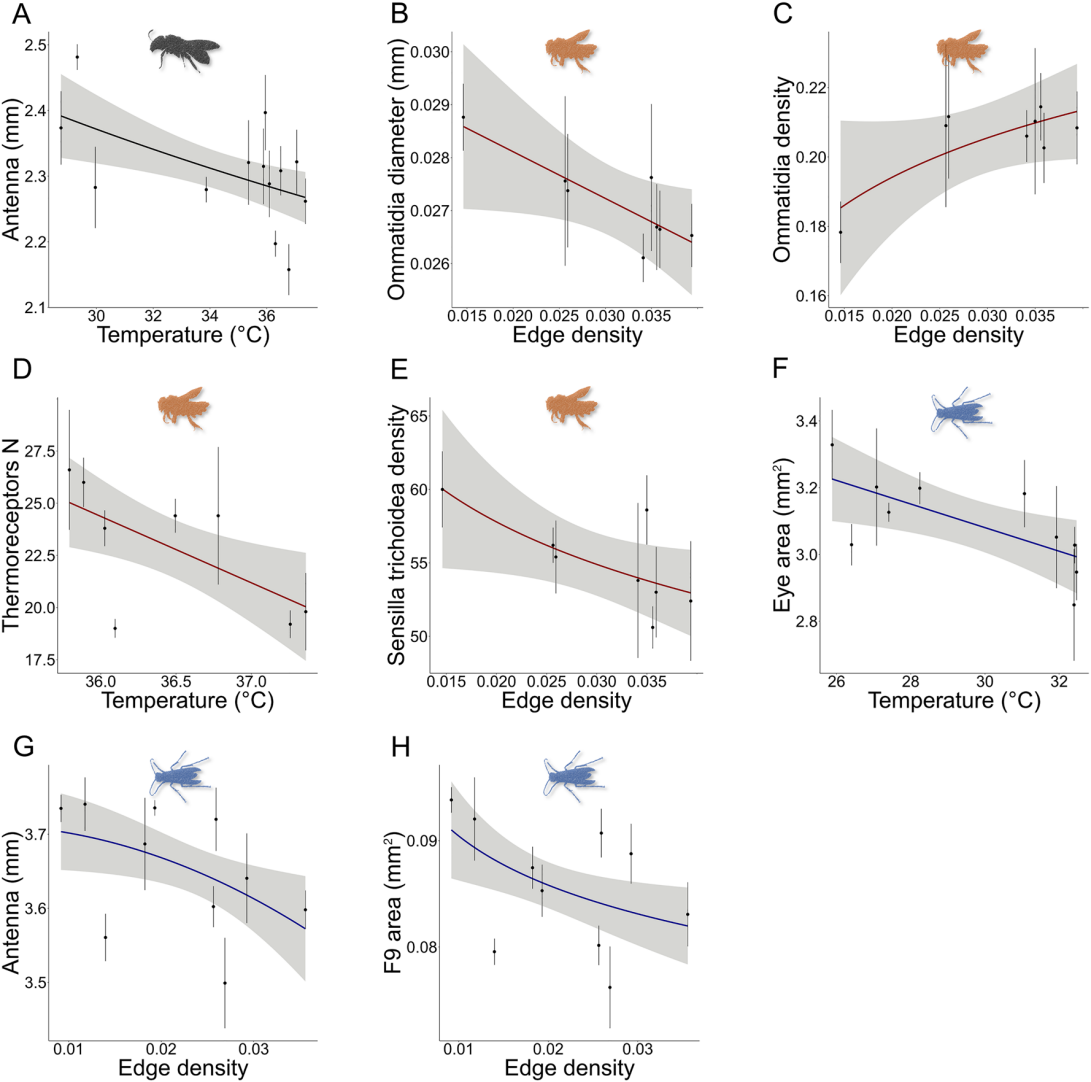
Plots of the significant linear mixed models used to test the effect of urbanisation on the morphometric parameters of the sensory systems. The mean value ± standard error is represented for each site. In grey, the 95% confidence interval. The colour of the line is associated with the species: *H. scabiosae* in black, *O. cornuta* in red, *P. dominula* in blue.

The Linear Mixed Models revealed species-specific responses of the measured traits to the two variables linked to urbanisation, though sometimes with a marginal significance (*P* < 0.09). In *H. scabiosae* we could not find any significant variations in the visual sensory system either with temperature or green fragmentation. However, we found *H. scabiosae* individuals having smaller antennae in hotter sites (Table 1, Fig. 4A). In *O. cornuta,* we found a significant reduction in ommatidia diameter (Table 1, Fig. 4B) and a marginally significant increase in their density (Table 1, Fig. 4C) in more fragmented sites. In this species, we also found a marginally significantly reduction in the number of thermoreceptors in hotter sites (Table 1, Fig. 4D) and a statistically significantly reduction in sensilla trichoidea in more fragmented areas (Table 1, Fig. 4E). In *P. dominula*, we found individuals with statistically significantly smaller eyes at warmer locations (Table 1, Fig. 4F) as well as with smaller antennae (Table 1, Fig. 4G) and F9 (Table 1, Fig. 4H) in more fragmented sites. The F9 of *P. dominula* was also marginally smaller in hotter sites (Table 1).

**Table 1:** Summary statistic of the linear mixed models used to analyse variations in the morphometric parameters of the sensory system along the urbanisation gradient.

Species	Trait	d.f	*N*	Predictors	Estimate	Statistic	*P*
*H. scabiosae*	Eye area	60	65	ED	2.545	1.439	0.155
				**ITD**^**2**^	0.152	15.545	** < 0.001**
	Ocellus diameter	60	65	ED	0.001	0.006	0.995
				**ITD**	0.062	12.179	** < 0.001**
	Interommatidial angle	60	65	ED	− 1.219	− 0.624	0.535
				**ITD**	− 0.440	− 6.804	** < 0.001**
	Ommatidia density	60	65	ED	− 0.021	− 0.055	0.956
				**ITD**	− 0.032	− 2.718	**0.009**
	Ommatidia diameter	60	65	ED	8.475	0.489	0.627
				**ITD**	1.959	3.167	**0.002**
	Antenna length	60	65	**Temp [Log]**	− 337.510	− 2.261	**0.025**
				ED	1637.159	1.100	0.242
				**ITD**	357.855	10.690	** < 0.001**
	F9 area	60	65	Temp [Log]	− 11,844.690	− 1.553	0.126
				ED	42,065.309	0.586	0.560
				**ITD**	2922.884	7.721	** < 0.001**
	PO number	41	46	Temp	0.036	0.131	0.896
				**ITD**	8.041	2.860	**0.007**
	SP density	59	64	Temp	− 0.025	− 0.209	0.835
				**ITD**	− 4.852	− 3.680	**0.001**
	ST density	59	64	Temp	0.396	0.815	0.419
				ITD	0.462	0.106	0.916
*O. cornuta*	Eye area	35	40	ED	2.520	0.350	0.728
				**ITD**	0.112	10.411	** < 0.001**
	Ocellus diameter	35	40	ED	0.541	1.319	0.196
				**ITD**	0.051	6.979	** < 0.001**
	Interommatidial angle	35	40	ED^2^	− 116.063	− 1.601	0.118
				ITD	− 0.076	− 0.979	0.335
	Ommatidia density	35	40	ED [Log]	0.022	1.679	0.088*
				**ITD**	− 0.060	− 4.881	** < 0.001**
	Ommatidia diameter	35	40	**ED**	− 73.983	− 2.063	**0.047**
				**ITD**	3.624	4.243	** < 0.001**
	Antenna length	35	40	Temp	− 8.568	− 0.146	0.885
				**ITD**	577.650	10.452	** < 0.001**
	F9 area	35	40	Temp	− 2312.163	− 1.349	0.186
				**ITD**	19,503.463	6.316	** < 0.001**
	PO number	35	40	Temp	− 3.124	− 1.960	0.058*
				ITD	0.574	0.274	0.786
	SP density	35	40	ED [Log]	3.333	1.204	0.254
				ITD	0.599	0.264	0.801
	ST density	35	40	**ED [Log]**	− 4.492	− 2.519	**0.016**
				**ITD**	− 6.532	− 2.034	**0.050**
*P. dominula*	Eye area	47	42	**Temp**	− 0.037	− 3.036	**0.004**
				**ITD**	0.114	3.666	**0.001**
	Ocellus diameter	47	42	ED	0.114	0.493	0.624
				**ITD**	0.048	4.139	** < 0.001**
	Interommatidial angle	47	42	Temp	0.004	0.592	0.557
				ITD	− 0.126	− 1.384	0.174
	Ommatidia density	47	42	Temp	0.001	0.772	0.444
				ITD	− 0.034	− 1.813	0.077*
	Ommatidia diameter	47	42	ED	12.752	0.410	0.684
				ITD	1.636	1.581	0.121
	Antenna length	47	42	**ED**^**2**^	− 121,449.014	− 2.430	**0.019**
				**ITD**	410.086	5.875	** < 0.001**
	F9 area	47	42	Temp [Log]	− 30,550.825	− 1.954	0.058*
				**ED [Log]**	− 7868.021	− 2.353	**0.023**
				**ITD**	12,889.429	2.185	**0.035**
	PO number	47	42	Temp	0.249	1.366	0.179
				ITD	− 1.413	− 0.606	0.548
	SP density	47	42	Temp	0.052	0.325	0.746
				ITD	− 3.759	− 1.814	0.077*
	ST density	47	42	Temp [Log]	49.877	1.548	0.129
				ITD	− 1.540	− 0.106	0.916
	SB density	46	41	ED	47.171	0.846	0.403
				ITD	− 4.220	− 1.881	0.067*

ITD: intertegular distance, F9: size of 9th flagellomere, PO: pit organs, SP: sensilla placoidea, ST: sensilla trichoidea, SB: sensilla basiconica, Temp: temperature, ED: edge density. d.f.: degrees of freedom, N: number of samples, *P*: *P*-value. In bold significant results, * marginally significant results.

## Discussion

Here, we presented the first study on the effects of urbanisation on some morphological aspects of the sensory system of three biologically distinct aculeate Hymenoptera. We were able to highlight some interesting variations in the studied morphometric parameters across a local temperature or green fragmentation gradients produced by urbanisation. We believe that our results are robust, as we took into account the intra-population variability of these traits, as well as body size, which is important in explaining variations in sensory system. In some cases, the results are in line with our hypotheses and are in agreement with what previously found in non-urban contexts. Overall, for the visual sensory system, we found significant variations in *O. cornuta* and *P. dominula*, but not in *H. scabiosae*. Such variable response is somehow in line with literature: for example, studies on the effect of temperature on visual sensory system has yielded contrasting results^34,61^. As for the antennal sensory system, we found significant variations in all the species, most of which in line with what found in the very few available previous studies^32^. However, important differences in the tested responses appeared among the three studied species.

### Visual system

In *H. scabiosae*, the variation in visual sensory system was only explained by body size and not environmental variables. This is in line with what found in ants reared at different temperatures which did not show variations in the ommatidium diameter^61^. Similarly, bumblebees reared at different temperatures did not show any difference between the mean eye area^62^. Interestingly, *H. scabiosae* is the species in which the body size has the strongest effect on the visual morphometric parameters. Thus, the fact that we could not find any significant variation in the visual sensory system might be due to the intrinsic morphology of this species. In fact, this species has proportionally—in relation to body size—higher values of ocellus diameter, ommatidia density, interommatidial angle and ommatidia diameter of the three species (Fig. S2); as well as having proportionally bigger eyes than *O. cornuta* (Fig. S2). These characteristics seem to suggest that the visual system in this species is very sharp and performant^17,19^, and therefore possibly less affected by environmental changes.

In other cases, higher temperatures were seen to affect the visual system. ^34^ found smaller eyes in *Drosophila* reared at higher temperatures. This is in line with what we found in *P. dominula*, which showed smaller eyes in hotter (i.e., more urbanised) areas*.* One possible explanation is that temperature influences epidermal cell size since at higher temperatures small cells are favoured according to the theory of optimal cell size (TOCS)^63,64^. In fact, higher temperatures require a higher demand for oxygen which favours tissues built of small cells, with an extensive network of cellular membranes for oxygen distribution^65^. Thus, considering that the area of the eye in *P. dominula* is positively correlated with ommatidia diameter, reduced eye size in hotter sites may be explained by the TOCS, as shown for an *Osmia* species^30^. However, it could be also hypothesised that wasps’ eyes develop faster in smaller individuals due to higher developmental temperatures^66^. This is somehow supported by the fact that in this species we found a strong inverse allometric relationship between body and eye size. That is, larger insects have proportionally smaller eyes that what predicted by body size in an isometric relationship. A similar allometric response, albeit regarding head size, was also found at higher temperatures in an *Isodontia* species^66^. Considering all the possible explanations, the reduction in eye size is somewhat of a handicap for the wasp. In fact, eye size is directly related to the visual resolution^17,19^. So that smaller eyes may be seen a disturbing effect of hotter temperatures rather than an adaptation.

In *O. cornuta*, we found that landscape composition rather than temperature is the most likely driver of variations in the visual sensory system. According to our hypothesis, we found a higher ommatidia density in more fragmented sites (i.e., more urbanised). Interestingly, we found edge density and not temperature to significantly explain variation in ommatidia size as well^30^. Of course, ommatidial size and density are inevitably linked to each other. In fact, one can hypothesise that the increase in density is made possible only by a reduction in the size of each ommatidia. Or vice versa, the reduction in the size of ommatidia brings consequently the increase in their density. Albeit we cannot disentangle these two trends, it is interesting to note that they have different effects on vision properties. In fact, on one hand, a higher density of ommatidia gives the insect a better visual resolution, while on the other, smaller ommatidia reduce the ability to capture light^19^. Our results are in contrast with those found in a butterfly species that showed larger ommatidia in naturally fragmented habitats^31^. However, the increase in ommatidia density may overcome the reduction in ommatidia size by increasing visual resolution. In fact, if the size is reduced, more ommatidia will fit into an eye of the same size, thus improving the density of spatial sampling of the image and therefore spatial resolution^67^. In *O. cornuta*, we also found that a reduced ommatidia size is correlated with smaller interommatidial angles, another possible way to increase visual resolution. It is also worth noting that *O. cornuta* has proportionally smaller ommatidia density and diameter—in relation to body size—which may be associated with poor visual properties^17,19^. These seemingly unfavourable physiological conditions might explain why in this species habitat fragmentation appears to be a driver of potential adaptive responses to the environment.

### Antennal system

As for the antennal sensory system, in *H. scabiosae* and *P. dominula*, we found smaller antenna in hotter and more fragmented areas, and smaller 9th flagellomere in more fragmented sites only in *P. dominula*. This seems in contrast with the Allen’s rule: the warmer the climate, the longer the appendages^68^. However, our results agree with what found in a butterfly species and in a bumblebee species, where individuals reared at higher temperatures result in adults with shorter antennae^35,36^. However, it was shown that in bees and wasps higher temperatures reduce body size either by accelerating the larval development or as an adaptation to reduce the risk of overheating^16^. Such tendency was also found in our studied species at the study metropolitan area^44^. Albeit with different drivers, it seems that in both species, urban areas host insects with smaller antennae.

As we proposed before, it could be hypothesised that in these species, antennae develop faster in smaller individuals due to higher developmental temperatures found in cities. Generally, temperature affects cell size rather than the number of cells according to the TOCS^60,61^. Thus, a decrease in cell size due to higher temperatures could explain the smaller morphological traits. In addition, a possible explanation may be found in the strong inverse allometric relationship between body size and the length of the antennae. In fact, antennal length was shown to be tightly coupled to body size no matter what developmental temperature the individual experiences^35^. At least for *P. dominula*, we think that the reduction in F9 area is due to the reduction in the antenna length, and that maybe this latter structure is the one under the effects of environmental change. As the size of sensory organs is typically related to their sensitivity^69^, smaller antennae in *P. dominula* and *H. scabiosae* in a more urban matrix may affect foraging efficiency, colony development and ultimately fitness.

Finally, we found that *O. cornuta* showed no variation in antennal length with temperature, similarly to *Bombus terrestris* (Linnaeus, 1758)^62^. However, interestingly, we found that *O. cornuta* has less thermoreceptors in hotter sites (i.e., more urbanised). The reduction in thermoreceptors was also found across larger temperature gradients and confirms our hypotheses^32^. In fact, the antennae of most insects, including bees, only possess receptors excited by cold and inhibited by heat^70^. Bees need to proper feel air temperature as it affects their flight activities^71^. It may be expected that outside cities bees experience colder ambient temperatures and so they might show more of these thermal receptors^72^. We also found less sensilla trichoidea in more fragmented areas (i.e., more urbanised). Since we found that the number of these two types of sensilla are positively correlated, it may be hypothesised that the drivers pushing towards a reduction in the number of thermoreceptors would affect the number of sensilla trichoidea as well. Nonetheless, this result is somehow in line with what found by^33^ in *Triatoma* bugs, where they found that specimens of the urban population showed a high phenotypic variability and that individuals raised under stable conditions of temperature had a pedicel with fewer sensilla. In cities, hotter sites are subjected to stronger heat waves, which may be a possible explanation of the increased number of sensilla trichoidea we found. Overall, we can hypothesise that a certain degree of morphological variability among populations may be associated with adaptations based on the sensory requirements of different urban conditions^33^.

## Conclusions

In conclusion, we reported for the first time the effects of urbanisation on the visual and antennal sensory systems of three biologically different Hymenoptera species. Despite possible shortcomings due to the reduced sample size and a marginal significance for some responses, we believe that our results are reasonably robust, given that we controlled for body size, which is a very strong driver of variation in the visual and antennal sensory systems. Our study highlights how changes in temperature and landscape configuration lead to intraspecific changes in morphological traits that may influence the perception of the environment. However, whether these differences would be adaptive or not remains to be tested. Nonetheless, we hypothesise that, at least here, the highlighted variation in the functional traits of the sensory system is likely due to phenotypic plasticity, as cities are rapidly changing environments and temperatures may shift from year to year. This was also suggested by^73^ for other phenotypic traits (i.e., body size) in bees observed across urbanisation gradients. This underlines how urbanisation may affect bees and wasps in a much broader way then previously hypothesised. This may have consequences on the conservation status of such insects in urbanised areas since the perception of the environment has inevitable impacts on the fitness as well. Further studies would certainly be needed to investigate if such variations occur also in other species or in other urban settings. Most importantly, the biological consequences behind the observed variations are likely to be elucidated by studies using a more behavioural and physiological approach, as well as fitness estimations.

### Supplementary Information

Supplementary Information 1.

Supplementary Figure S1.

Supplementary Figure S2.

Supplementary Information 4.

## Data Availability

All data generated or analysed during this study are included in the Supplementary Information files.
