# Supplementary figures and images for: Subtle morphological changes in the visual and antennal sensory system of bees and wasps across an urbanisation gradient
Source: Sci Rep. 2024 Apr 18;14:8960. doi: 10.1038/s41598-024-58804-2 (PMC11026482; doi:10.1038/s41598-024-58804-2)

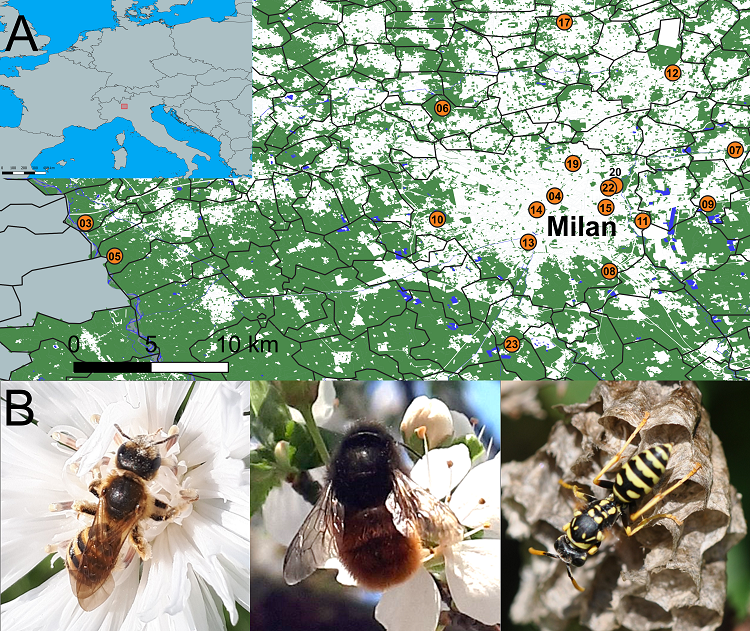

Supplement: Supplementary file 2 — Supplementary Figure S1. [file 41598_2024_58804_MOESM2_ESM.tif]

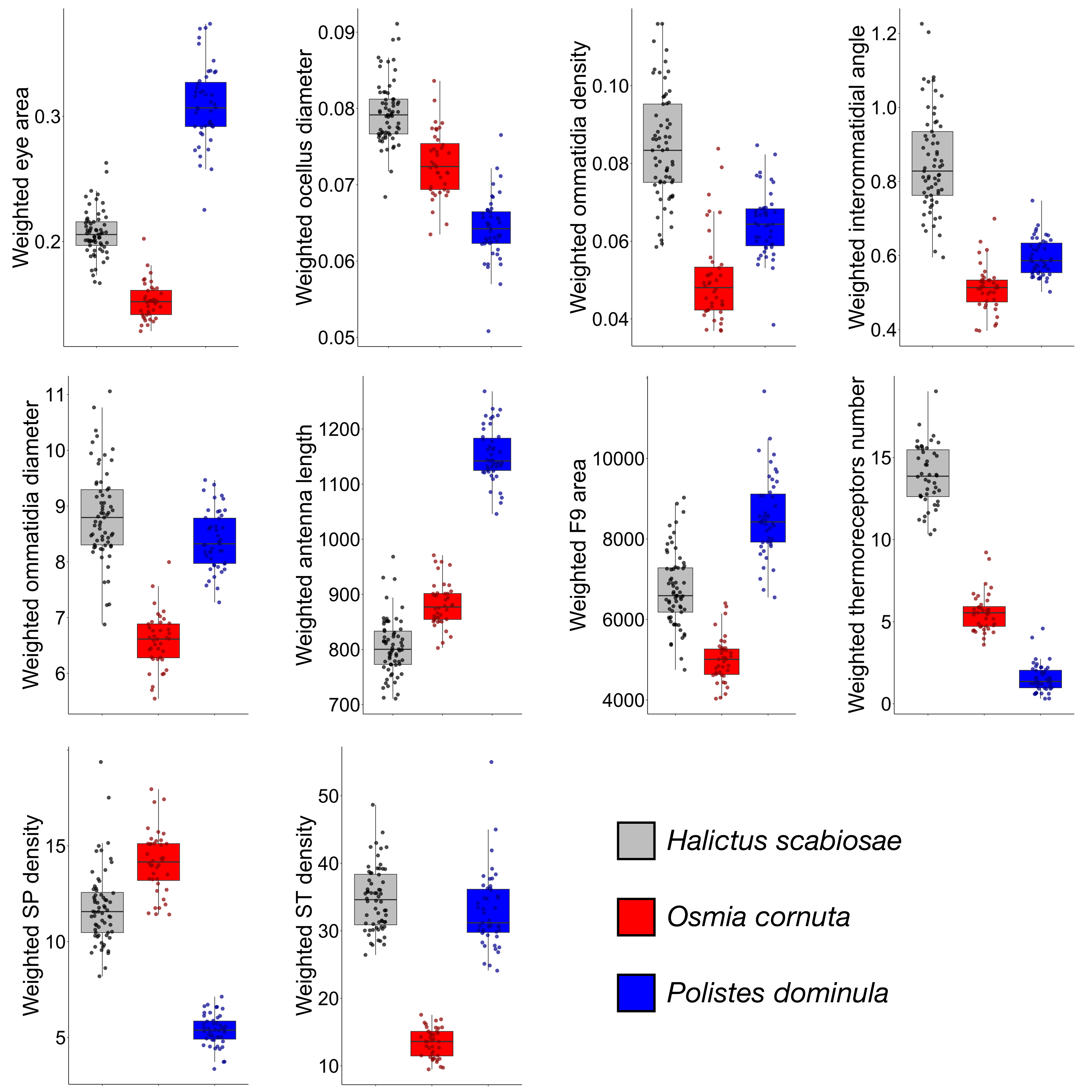

Supplement: Supplementary file 3 — Supplementary Figure S2. [file 41598_2024_58804_MOESM3_ESM.tif]
