# Supplementary material for: Subtle morphological changes in the visual and antennal sensory system of bees and wasps across an urbanisation gradient
Source: Sci Rep. 2024 Apr 18;14:8960. doi: 10.1038/s41598-024-58804-2 (PMC11026482; doi:10.1038/s41598-024-58804-2)
Supplement: Supplementary file 4 — Supplementary Information 4. [file 41598_2024_58804_MOESM4_ESM.docx]

**Supplementary information (supplementary tables and figure legends) for the manuscript:**

Subtle morphological changes in the visual and antennal sensory system of bees and wasps across an urbanisation gradient

Andrea Ferrari^1^*, Greta Tacconi^1^, Carlo Polidori^1^

^1^ Department of Environmental Sciences and Policies (ESP), University of Milan, via Celoria 26, 20133, Milan (Italy)

***Corresponding author:** Andrea Ferrari, email: andrea.ferrari@unimi.it

**Table S1.** List of the environmental variables describing each sampling site. T: temperature, E.D.: edge density, G/I: ratio between green (G) and impervious (I) surfaces. N: individuals analysed for each site; “-“ means no specimens of that species were found in that site.

|  |  |  | *Halictus scabiosae* | | | *Osmia cornuta* | | *Polistes dominula* | | | For all species | |
| --- | --- | --- | --- | --- | --- | --- | --- | --- | --- | --- | --- | --- |
|  |  | **Larval development period** |  | April-May 2022 | May-June 2022 |  | April-August 2021 |  | April 2022 | May 2022 |  |  |
| **Site ID** | **Latitude** | **Longitude** | **N** | **T(°C)** | **T(°C)** | **N** | **T(°C)** | **N** | **T(°C)** | **T(°C)** | **G/I** | **E.D.** |
| 23 | 45.362288 | 9.157196 | 5 | 28.452 | 33.870 | - | 33.870 | - | 26.901 | 31.649 | 1.014 | 0.035 |
| 03 | 45.462316 | 8.805235 | 5 | 25.253 | 29.961 | - | 29.961 | - | 23.575 | 27.228 | 44.798 | 0.007 |
| 04 | 45.484590 | 9.192524 | 5 | 29.973 | 35.886 | 5 | 35.886 | - | 27.695 | 32.338 | 0.110 | 0.035 |
| 15 | 45.475239 | 9.234793 | 5 | 29.964 | 36.095 | 5 | 36.095 | - | 27.785 | 32.635 | 0.017 | 0.034 |
| 19 | 45.511397 | 9.207611 | 5 | 29.666 | 37.059 | 5 | 37.059 | - | 27.363 | 32.674 | 0.063 | 0.036 |
| 17 | 45.628176 | 9.200449 | 5 | 29.190 | 37.362 | - | 37.362 | 5 | 27.261 | 32.401 | 0.800 | 0.027 |
| 05 | 45.435017 | 8.829432 | - | 26.405 | 29.910 | - | 29.910 | 5 | 24.505 | 28.245 | 91.106 | 0.009 |
| 07 | 45.522613 | 9.341480 | 5 | 29.152 | 35.951 | - | 35.951 | 5 | 27.079 | 32.012 | 0.169 | 0.029 |
| 08 | 45.422497 | 9.237726 | 5 | 28.446 | 35.359 | - | 35.359 | 5 | 26.957 | 31.065 | 2.695 | 0.019 |
| 09 | 45.478127 | 9.318729 | - | 29.405 | 36.201 | - | 36.201 | 5 | 27.499 | 32.419 | 1.926 | 0.012 |
| 10 | 45.465510 | 9.095742 | 5 | 28.571 | 36.495 | 5 | 36.495 | 4 | 26.404 | 31.713 | 0.996 | 0.026 |
| 06 | 45.557056 | 9.099943 | - | 28.002 | 35.792 | 5 | 35.792 | 5 | 25.884 | 31.052 | 3.076 | 0.026 |
| 11 | 45.463942 | 9.265182 | 5 | 29.324 | 36.903 | - | 36.903 | 4 | 27.136 | 32.469 | 0.174 | 0.014 |
| 20 | 45.493294 | 9.242187 | - | 29.835 | 36.187 | - | 36.187 | 3 | 27.409 | 32.573 | 0.752 | 0.018 |
| 12 | 45.586167 | 9.290016 | 5 | 28.751 | 37.259 | 5 | 37.259 | 5 | 26.580 | 31.926 | 0.135 | 0.036 |
| 13 | 45.446457 | 9.170732 | 5 | 29.689 | 36.780 | 5 | 36.780 | - | 27.851 | 33.162 | 0.195 | 0.039 |
| 14 | 45.473228 | 9.177705 | - | 29.763 | 36.027 | 5 | 36.027 | - | 27.343 | 32.720 | 0.101 | 0.014 |
| 22 | 45.491122 | 9.236950 | 5 | 30.130 | 36.306 | - | 36.306 | - | 27.724 | 32.871 | 0.005 | 0.018 |

**Table S2.** Descriptive statistics of the morphometric parameters analysed (mean ± standard error).

| **Morphological trait** | ***H. scabiosae*** | ***O. cornuta*** | ***P. dominula*** |
| --- | --- | --- | --- |
| Intertegular distance (mm) | 2.879 ± 0.004 | 4.136 ± 0.008 | 3.174 ± 0.004 |
| Head width (mm) | 3.552 ± 0.005 | 4.296 ± 0.007 | 3.715 ± 0.003 |
| Eye area (mm^2^) | 1.710 ± 0.004 | 2.605 ± 0.009 | 3.096 ± 0.006 |
| Ocellus diameter (mm) | 0.228 ± 0.0003 | 0.300 ± 0.0005 | 0.204 ± 0.0003 |
| Ommatidia density | 0.240 ± 0.0004 | 0.205 ± 0.0008 | 0.204 ± 0.0005 |
| Interommatidial angle | 2.523 ± 0.003 | 2.103 ± 0.004 | 1.882 ± 0.002 |
| Ommatidia diameter (μm) | 25.289 ± 0.021 | 27.163 ± 0.053 | 26.557 ± 0.026 |
| Antenna length (μm) | 2306.441 ± 0.021 | 3639.867 ± 5.470 | 3652 ± 2.398 |
| F9 area (μm^2^) | 55387.309 ± 98.916 | 85152.581 ± 221.479 | 85762.673 ± 164.540 |
| Thermoreceptors | 39.522 ± 0.119 | 22.900 ± 0.116 | 4.915 ± 0.055 |
| Sensilla placoidea | 33.516 ± 0.047 | 58.100 ± 0.119 | 17.000 ± 0.048 |
| Sensilla trichoidea | 98.813 ± 0.136 | 55.000 ± 0.171 | 104.128 ± 0.0343 |
| Sensila basiconica | - | - | 14.065 ± 0.053 |

**Supplementary figure legends**

**Fig. S1.** A) Map of the sampling sites. The left panel shows the location of the study area (Metropolitan city of Milano) in Europe. In the map, orange points represent the sampling sites with the ID code (see table S1). In green vegetated areas, in white cemented surfaces, in blue water. Municipalities borders are shown with the black solid line. B) Pictures taken on field of females of the three studied species. Map created in QGIS 3.26.3 (<https://qgis.org/en/site/>).

**Fig. S2.** Boxplot showing the weighted by intertegular distance values of the visual and antennal morphological parameters. Dots shows actual values, boxes shows mean (horizontal line) first and third quartiles as margins of the boxes.
